# Microparticles from septic shock patients contain microRNA and messenger RNA: new players in the pathogenesis of sepsis?

**DOI:** 10.1186/cc12995

**Published:** 2013-11-05

**Authors:** Luciano CP Azevedo, Juliana M Real, João E Bezerra, Flavia R Machado, Reinaldo Salomao

**Affiliations:** 1 Instituto Sírio-Libanês de Ensino e Pesquisa, São Paulo, Brazil; 2 State University of São Paulo, Brazil; 3 Federal University of São Paulo, Brazil

## Background

Previous studies demonstrated the presence of microparticles (exosomes) in plasma of septic patients. These are cell-derived vesicles containing specific collections of proteins, lipids and genetic material that participate in the intercellular communication, changing the function and physiology of their target cells. The role of exosomes in sepsis, however, remains deeply unexplored. This study aimed to investigate the composition of microRNAs and messenger RNAs related to inflammatory response in circulating microparticles of septic shock patients.

## Materials and methods

Fourteen patients had 30 ml blood collected in the first 48 hours of sepsis and 7 days after for those who survived. Five healthy volunteers served as controls. Exosomes were isolated from plasma by filtration (0.22 μM) and ultracentrifugation. Thirty nanograms of the total RNA were reversely transcribed and the expression profile of 754 human miRNAs and 91 mRNAs from immune response was evaluated by real-time quantitative PCR using the Taqman Low Density Array (Applied Biosystems). The raw data were processed in Expression Suite v1.0.1 software and analyzed in StatMiner v3.0 software considering the global expression level for normalization. The fold-change was calculated based on the estimated mean difference (2^(-ΔCT)^).

## Results

Different miRNA expression was observed in the exosomes from septic patients in comparison with healthy donors. In the first 48 hours of septic shock, three miRNAs were differentially expressed: miR-1290 (2.78-fold, *P *= 0.02), miR-1298 (4.02-fold, *P *= 0.03) and miR-146a (-2.51-fold, *P *= 0.02). In the recovery phase of sepsis, five miRNAs were differently expressed as compared with controls: miR-1260 (2.29-fold, *P *= 0.02), miR-1274A (2.83-fold, *P *= 0.02), miR-1274B (3.31-fold, *P *= 0.02), miR-192 (1.83-fold, *P *= 0.02) and miR-604 (-6.41-fold, *P *= 0.02). The miRNA expression profiles in different stages of sepsis were similar. Moreover, exosomes from patients after 1 week of sepsis carry less CCL5 mRNA than in the beginning of the disease (-2.49-fold, *P *= 0.02).

## Conclusions

Exosomes from septic shock patients carry different miRNA expression profiles at different stages of the disease, as compared with healthy individuals. CCL5 mRNA is less expressed in the recovery phase of sepsis. The composition of these vesicles may help to understand the underlying mechanisms involved in their role in the pathogenesis of sepsis.

